# Current status of laparoscopic total gastrectomy

**DOI:** 10.1002/ags3.12208

**Published:** 2018-09-17

**Authors:** Yoshihiko Kawaguchi, Kensuke Shiraishi, Hidenori Akaike, Daisuke Ichikawa

**Affiliations:** ^1^ First Department of Surgery Faculty of Medicine University of Yamanashi Chuo Yamanashi Japan

**Keywords:** esophagojejunostomy, gastric cancer, laparoscopic total gastrectomy, lymphadenectomy, postoperative complication

## Abstract

In this article, the current state of laparoscopic total gastrectomy (LTG) was reviewed, focusing on lymph node dissection and reconstruction. Lymph node dissection in LTG is technically similar to that in laparoscopic distal gastrectomy for early gastric cancer; however, LTG for advanced gastric cancer requires extended lymph node dissections including splenic hilar lymph nodes. Although a recent randomized controlled trial clearly indicated no survival benefit in prophylactic splenectomy for lymph node dissection at the splenic hilum, some patients may receive prognostic benefit from adequate splenic hilar lymph node dissection. Considering reconstruction, there are two major esophagojejunostomy (EJS) techniques, using a circular stapler (CS) or using a linear stapler (LS). A few studies have shown that the LS method has fewer complications; however, almost all studies have reported that morbidity (such as anastomotic leakage and stricture) is not significantly different for the two methods. As for CS, we grouped various studies addressing complications in LTG into categories according to the insertion procedure of the anvil and the insertion site in the abdominal wall for the CS. We compared the rate of complications, particularly for leakage and stricture. The rate of anastomotic leakage and stricture was the lowest when inserting the CS from the upper left abdomen and was significantly the highest when inserting the CS from the midline umbilical. Scrupulous attention to EJS techniques is required by surgeons with a clear understanding of the advantages and disadvantages of each anastomotic device and approach.

## 1 INTRODUCTION

Gastric cancer is the fifth most common malignancy and the third leading cause of cancer death in the world.^1^ Although various new drugs have been developed for its treatment, surgically curative resection is still the mainstay of treatment for gastric cancer. Since the first laparoscopic gastrectomy case was reported in 1991,^2^ it has gained widespread global popularity owing to laparoscopic hemostatic surgical devices and the standardization of techniques. Several randomized controlled trials (RCT) comparing laparoscopic distal gastrectomy (LDG) with conventional open distal gastrectomy (ODG) have reported superiority in the short‐term advantages for LDG.^3^, ^4^, ^5^, ^6^, ^7^, ^8^, ^9^, ^10^ Although RCT are ongoing in both Japan and Korea (JCOG0912^11^ and KLASS01^12^), several large‐scale retrospective studies have shown acceptable prognostic results of LDG for patients with early gastric cancer.^13^, ^14^, ^15^, ^16^, ^17^ In recent years, LDG has been implemented in patients with advanced gastric cancer at high‐volume centers, and RCT comparing the feasibility and long‐term survival between LDG and ODG are currently ongoing in China (CLASS‐01 trial^18^), Korea (KLASS‐02 trial^19^), and Japan (JLSSG0901^20^).

In contrast, laparoscopic total gastrectomy (LTG) is not common compared with LDG, which is carried out in only 25% (1556/6183) of total gastrectomy procedures, according to a questionnaire‐based survey conducted by the Japan Society of Endoscopic Surgery in 2015, although the proportion of LDG had increased to 54% (6884/12 722).^21^ In this survey, the conversion rate in LTG was reported as 2.1% which was about three times as compared with 0.6% in LDG. Furthermore, according to the National Clinical Database (NCD), covering 95% of general surgery procedures in all of Japan, LTG is carried out in 18% (5749/32 144) of total gastrectomy procedures.^22^

Several reports have already published data on the feasibility and safety of LTG, but these reports were mainly from high‐volume centers, and almost all the LTG were carried out by surgeons who were already accustomed to laparoscopic gastrectomy. Many surgeons still hesitate to carry out LTG, and the main reasons are difficulty of lymphadenectomy at the splenic hilum and the high technical demands of esophagojejunostomy (EJS).

Two large‐scale reports based on data from the National Clinical Database, one retrospective^22^ and the other prospective,^23^ have been recently reported as so‐called “real‐world data” in Japan, with controversial results about the occurrence of anastomosis‐related complications.

In this article, the status of LTG was reviewed focusing on lymphadenectomy and reconstruction.

## 2 LYMPHADENECTOMY

Lymphadenectomy, excision of the regional lymph nodes (LN) draining from a tumor, is an essential element in the surgical management of gastric cancer. The extent of systematic lymphadenectomy is defined, respectively, for each type of gastrectomy, according to Japanese gastric cancer treatment guidelines.^24^ In principle, D1 and D1 + lymphadenectomy is indicated for early gastric cancer, and D2 lymphadenectomy for advanced gastric cancer and cases with apparent metastasis at the regional lymph nodes, even in early gastric cancers. In D1 + lymph node dissection, differences between LDG and LTG are only left paracardial LN (No. 2), left greater curvature LN along the short gastric arteries (No. 4sa) which is usually easily removed with the stomach in LTG, and proximal splenic artery LN (No. 11p) which is generally removed in LDG for early gastric cancer. In that sense, technical aspects of lymph node dissection in LTG are like those in LDG for early gastric cancer; therefore, the prognostic evidence based on results of RCT (JCOG 0912), which confirm that LDG is not inferior to ODG in efficacy for early gastric cancer, would be applicable to LTG.

However, LTG for advanced gastric cancer requires precise lymph node dissection. As for LDG, two RCT, JLSSG0901 and KLASS‐02, are in progress and the results will soon be available; they compare long‐term outcomes of LDG with those of ODG. In contrast, although some retrospective studies have reported that long‐term outcome of LTG is equivalent to that of open total gastrectomy (OTG), RCT in LTG for advanced gastric cancer has just been started in Korea (KLASS‐06).

Among several LN stations, splenic hilar (No. 10) and along the distal splenic artery (No. 11d), the LN are specific and are the most applicable regions for the procedure of LTG for advanced gastric cancers. Survival benefit of lymphadenectomy for these regions remains controversial not only for laparoscopic surgery, but also for open surgery of advanced gastric cancer.

The difficulty of lymphadenectomy of the region is due to anatomical variation of the splenic hilar vessels and the narrow and deep space, and lymphadenectomy increases the risks of operative morbidities including pancreatic fistula. In recent reports, the incidence of pancreatic fistula in LTG ranged between 0.2% and 2.7% and this rate is equivalent to OTG.^22^, ^23^, ^25^, ^26^ Furthermore, some reports^22^, ^25^ showed that the incidence of overall complications of LTG was equivalent to that of OTG, and other reports showed that the rate of complications was lower for LTG than for OTG.^23^, ^26^, ^27^ One of the reasons for this is the improvement of energy devices, which is described in many reports,^28^, ^29^ and some researchers reported that preoperative assessment of splenic vascular anatomy using computed tomography (CT) with 3‐D imaging was useful and correlated with shorter operative time, lower blood loss,^30^ and a larger number of retrieved lymph nodes.^31^

Open total gastrectomy with splenectomy has been standard in Japan for complete removal of lymph nodes at the splenic hilum. Splenectomy can be done safely even in laparoscopic surgery by experienced surgeons, and the procedure itself is feasible with good short‐term outcomes.^32^, ^33^, ^34^ Several recent retrospective reports, however, showed that splenectomy in open total gastrectomy could increase postoperative morbidity and mortality^35^, ^36^ without survival benefit.^37^, ^38^ In 2017, a multi‐institutional RCT comparing splenectomy with spleen preservation in proximal gastric cancer was conducted in Japan.^39^ Splenectomy was associated with higher morbidity and greater blood loss, but had no survival benefit. The RCT concluded that prophylactic splenectomy, even in open surgery, should be avoided not only for operative safety but also for survival benefit, except for cases with tumors invading the greater curvature and with Borrmann type 4 tumors (linitis plastica).

Conversely, the clinical significance of lymph node dissection for cases with tumors invading the greater curvature and type 4 advanced gastric cancer remains a matter of debate. We reported that patients with tumors localized on the greater curvature and type 4 cancer might obtain relatively high survival benefits from splenic hilar lymph node dissection.^40^ Son et al^41^ reported long‐term results of patients with splenic hilar lymph node metastasis treated by splenectomy (n = 258) or spleen‐preserving hilar lymph node dissection (n = 344). They compared the therapeutic index of splenic hilar lymph node dissection to that for LN dissection at other extraperigastric lymph nodes, such as anterosuperior LN along the common hepatic artery (No. 8a), celiac artery LN (No. 9) etc., and they demonstrated that both therapeutic efficacies were similar. From these findings, Son et al advocated that splenic hilar lymph node dissection is necessary to improve prognosis.

The only certainty at this point is that some patients may receive prognostic benefit from adequate splenic hilar lymph node dissection. Some experienced surgeons have reported laparoscopic techniques of spleen‐preserving splenic hilum lymphadenectomy (SPSL). Uyama et al^42^ first introduced a hand‐assisted technique for complicated SPSL. Most surgeons still adopt the suprapancreatic approach, and some experienced surgeons have reported improved techniques for SPSL with some modifications. Mou et al^43^ developed a modified approach (combined supra‐ and infra‐pancreatic approaches), and Huang et al^44^ developed a surgical procedure by following the perigastric fascia and the intrafascial space according to the embryological and anatomical background. They advocated that the new, safer approaches enabled exposure and dissection of splenic hilum lymph nodes in an approach posterior to the splenic artery. By several techniques described above, most articles have indicated that a laparoscopic approach could also obtain similar short‐term results concerning the number of retrieved splenic hilar lymph nodes and occurrence rates of postoperative complications. Further large‐scale study should be conducted to establish the clinical significance of splenic hilar lymph node dissections.

## 3 RECONSTRUCTIONS

In 1999, Azagra et al^45^ reported the first case of EJS reconstruction carried out by small laparotomy after LTG. Some other reports have demonstrated good short‐term results after EJS with circular stapler (CS) carried out through a small incision.^46^, ^47^, ^48^ However, inserting and fixing the anvil head into the esophageal stump is sometimes difficult in a narrow and deep operative field. Safe and secure anastomosis is difficult to carry out, requiring careful attention to avoid intervening in surrounding tissues between the anastomotic plane, especially in obese patients. Several intracorporeal EJS techniques have been developed as appropriate laparoscopic reconstruction methods after total gastrectomy. The techniques can be divided into two categories: those using a CS, and those using a linear stapler (LS).

A great advantage of the CS method is its familiarity in conventional open total gastrectomy. There are some other advantages of the CS method compared with the LS method, including no need for intracorporeal suturing procedures and longer trimming of the esophagus. In contrast, advantages of the LS method include better visual field during anastomosis and adaptability for intramediastinal anastomosis in cases with esophageal invasion.

Umemura et al^49^ reviewed 254 cases of the CS method and 729 cases of the LS method. They reported that the CS method was significantly associated with high rates of leakage (4.7% vs 1.1%, *P* < 0.001) and stenosis (8.3% vs 1.8%, *P* < 0.001) when compared with the LS method. However, the authors discussed that the complication rates partially depended on the experience of the surgeons. Inokuchi et al^50^ reviewed anastomotic complications in 46 case studies of LTG to compare various procedures for EJS. They classified anastomosis into six categories: (i) extracorporeal reconstruction by a single‐stapling technique using a CS; (ii) intracorporeal reconstruction by a single‐stapling technique using a CS; (iii) intracorporeal reconstruction by a double (or hemi‐double) stapling technique using a CS with a transabdominally inserted anvil; (d) intracorporeal reconstruction by a double (or hemi‐double) stapling technique using a CS with a transorally inserted anvil (Orvil, Medtronic plc, Dublin, Ireland); (e) intracorporeal reconstruction by side‐to‐side anastomosis using an LS; and (f) intracorporeal reconstruction by functional end‐to‐end anastomosis using LS. Inokuchi and colleagues reported that the incidence of EJS leakage was similar (1.1%‐3.2%), although the incidence of EJS stenosis was relatively high when the OrVil device was used (8.8%) compared with other procedures (1.0%‐3.6%). They discussed the use of a small anvil in some cases for easy passage through the esophageal entrance as the presumed cause of high rates of stenosis. Kyogoku et al^51^ reported that there was no difference in the postoperative complication rates related to the type of stapler when surgeons accredited through the Endoscopic Surgical Skill Qualification System of the Japanese Society of Endoscopic Surgery carried out EJS. The authors concluded that determination of the EJS procedure should be selected by preference and experience of the surgeon.

In Table 1, literature that compares two anastomosis methods in a single institution is shown.^51^, ^52^, ^53^, ^54^, ^55^, ^56^ Five of the studies reported that morbidity such as anastomotic leakage and stricture were not significantly different in CS and LS methods; however, one report showed that the LS method has fewer complications.

**Table 1:** Summary reported comparing circular stapler and linear stapler after total gasrectomy in same institution

Authors	No. of patients	Stapler	Method	Mean operative time (min)	Mean blood loss (ml)	Morbidity (%)	Leakage rate (%)	Stricture rate (%)	Mortality (%)	LOH (day)	Year
Kim EY^52^	29	CS	mini‐laparotomy	230.3	106.3	17.2	0	3.4	0	9.7	2016
	27	LS	linear	228.9	90.9	18.5	3.7	0	0	13.6	
Kawamura H^53^	49	CS	Orvil	259.5	53.3	8.2^a^	4.1	4.1^a^	NA	NA	2017
	139	LS	overlap	276.5	69.7	0.7^a^	0.7	0^a^	NA	NA	
Gong CS^54^	266	CS	mini‐laparotomy	170	NA	NA	5.6	1.1	NA	7	2017
	421	LS	FEEA	149	NA	NA	3.6	0.5	NA	6.8	
Yasukawa D^55^	51	CS	Orvil	346.1	34	9.8	3.9	0	0	13.0	2017
	18	LS	FEEA	348.4	35	5.6	0	5.6	0	12.0	
Kyogoku N^51^	83	CS	Orvil or mini‐lapatomy	330^a^	100^a^	25	4.0	7.0	NA	10^a^	2018
	208	LS	FEEA or overlap	297^a^	23^a^	20	1.0	5.0	NA	13^a^	
Yoshikawa K^56^	36	CS	Orvil	345^a^	45^a^	13.9	0	8.3	2.8	17.2	2018
	47	LS	overlap	398^a^	126^a^	10.6	4.3	0	0	19.5	

CS, circular stapler; FEEA, functional end?to?end anastomosis; LS, linear stapler; LOH, length of hospital stay; NA, not available.

Orvil, Medtronic plc, Dublin, Ireland.

a *P* < 0.05

## 4 LINEAR STAPLER

Esophagojejunostomy using LS is mainly divided into two types; a functional end‐to‐end anastomosis (FEEA) and a side‐to‐side anastomosis (called the “overlap method”).

Uyama et al^57^ first reported EJS using LS by a completely intra‐abdominal approach. Since then, the required knowledge and skills for the FEEA procedure have been reported in several papers. Although this method is simple, sufficient mobilization of the esophageal stump and the jejunal limb is required to reduce tension at the anastomotic site. Furthermore, side‐to‐side anastomosis is occasionally difficult as a result of the peri‐hiatal restricted space, especially in proximal cancer with esophageal invasion and esophagogastric junction cancer. Based on these weaknesses, Inaba et al^58^ reported another side‐to‐side anastomosis method called the “overlap method”. Furthermore, in recent years, several modified procedures of the overlap method have been reported. Nagai et al^59^ made the anastomosis in an inverted T‐shape to prevent the anastomosis from slipping into the mediastinum. Yamamoto et al^60^ transected the esophagus while being rotated by 90 degrees, making suturing of the entry hole easier. In either method, a 45‐mm cartridge is usually used in the anastomosis of the LS, and there is an advantage that the anastomosis diameter can be made larger as compared with the EJS using a CS.

Many papers have reported the safety and feasibility of EJS conducted by LS. However, the need for suturing technique and enough mobilization of the esophageal stump may sometimes cause nonexpert laparoscopic surgeons to hesitate about introducing EJS using the LS. Recent development and application of a barbed absorbable closure device (V‐Loc; Medtronic plc, Dublin, Ireland) may eliminate the hesitation about hand‐sewn suturing.^61^

Interestingly, esophageal hiatal hernia has recently been reported as a postoperative complication of EJS using LS after LTG. Ito et al^62^ reported that postoperative esophageal hiatal hernia occurred in seven (9%) of 78 patients who underwent LTG for gastric cancer, and all of them were patients who had an incision in the diaphragm. They concluded that when the crus was incised to improve the visual field of the anastomosis, it should have been repaired.

## 5 CIRCULAR STAPLER

As described above, many surgeons are familiar with reconstruction methods using the CS. Therefore, the CS method has been more widespread than the LS method, especially in the introductory period. Since the transorally inserted anvil (OrVil) was developed, it is easier and very convenient to carry out intracorporeal EJS and esophagogastrostomy. Several reports have reported the safety and feasibility of EJS carried out by the CS method in LTG.^63^ However, some papers noted a high incidence of postoperative complications in CS methods.

For this paper, a literature retrieval was carried out in PubMed for January 1, 1997 through April 30, 2018. The search terms included “laparoscopic,” “total gastrectomy,” and “gastric cancer.” Reports in languages other than English, reviews, and meta‐analyses were excluded, and cases <10 were also excluded.

We reviewed anastomotic complications, especially those reported on leakage and stricture, in 43 extracted studies of LTG.^33,46,48,52–56,63–97^ We compared the surgical results of various anastomotic procedures for EJS using the CS, with attention to the insertion procedure of the anvil and the insertion site of the anastomotic device.

First, the insertion procedure of the anvil was classified into the following three categories: (i) single‐stapling technique (SST) using hand‐sewn purse‐string suture or purse‐string instrument (PSI); (ii) double‐stapling technique (DST)/hemi‐double‐stapling technique (HDST) with transabdominally inserted anvil; or (iii) DST/HDST with transorally inserted anvil (OrVil) (Table 2).

**Table 2:** Summary reported of esophagojejnostomy methods after laparoscopic total gastrectomy for gastric cancer n (%)

Authors	Nation	Method	No. of patients	Morbidity	Leakage	Stricture	Year
1) SST							
Usui S^64^	Japan	PSI	15	NA	0 (0)	0 (0)	2008
Kinoshita T^65^	Japan	hand‐sewn	10	NA	0 (0)	0 (0)	2010
Lee JH^33^	South Korea	PSI	79	NA	2 (2.5)	0 (0)	2012
Shim JH^66^	South Korea	hand‐sewn	12	NA	2 (17)	5 (42)	2013
Kim HI^67^	South Korea	hand‐sewn	36	NA	0 (0)	0 (0)	2013
Yoshikawa T^68^	Japan	hand‐sewn	20	NA	0 (0)	0 (0)	2013
Du J^69^	China	hand‐sewn	52	NA	0 (0)	0 (0)	2014
Matsuda T^70^	Japan	hand‐sewn	21	3 (14.3)	1 (4.8)	1 (4.8)	2015
Kosuga T^71^	Japan	hand‐sewn	65	11 (16.9)	2 (3.1)	4 (6.2)	2015
Yamada T^73^	Japan	hand‐sewn	10	0 (0)	0 (0)	0 (0)	2015
Chen K^72^	China	hand‐sewn	18	5 (27.8)	1 (5.6)	1 (5.6)	2016
Amisaki M^74^	Japan	PSI	10	NA	0 (0)	0 (0)	2016
Kim EY^52^	South Korea	hand‐sewn	29	5 (17.2)	0 (0)	1 (3.4)	2016
Gong CS^54^	South Korea	minilapatomy	266	74 (27.8)	15 (5.6)	3 (1.1)	2017
Okuno K^75^	Japan	PSI	94	NA	0 (0)	2 (2.1)	2017
Liu W^76^	China	PSI	41	NA	0 (0)	0 (0)	2017
Total			778		23 (3.0)	17 (2.2)^a^	
2) DST/HDST with trans‐abdominally inserted anvil							
Omori T^77^	Japan	HDST	10	NA	0 (0)	0 (0)^a^	2009
Muguruma K^78^	Japan	HDST	32	NA	0 (0)	0 (0)^a^	2014
Zhao YL^79^	China	HDST	26	NA	0 (0)	0 (0)^a^	2014
Kim JH^80^	South Korea	DST	58	8 (13.8)	0 (0)	1 (1.7)^a^	2015
Ichikawa D^63^	Japan	HDST (lift up)	58	9 (15.5)	0 (0)	2 (3.4)^a^	2015
Wang H^81^	China	HDST	42	NA	1 (2.4)	2 (4.8)^a^	2015
Kosuga T^71^	Japan	HDST (lift up)	71	23 (32.4)	7 (9.9)	13 (18.3)^a^	2015
Ali B^82^	South Korea	HDST	58	NA	3 (5.2)	5 (8.6)^a^	2017
Li X^83^	China	HDST	24	3 (12.5)	1 (4.2)	0 (0)^a^	2017
Total			379		12 (3.2)	23 (6.1)^a^	
3) DST/HDST with trans‐orally inserted anvil (Orvil)							
Jeong O^84^	South Korea	Orvil	16	NA	0 (0)	0 (0)^a^	2009
Sakuramoto S^85^	Japan	Orvil	24	NA	0 (0)	0 (0)^a^	2010
Kachikwu EL^86^	United States	Orvil	16	NA	0 (0)	3 (18.8)^a^	2011
Kunisaki C^46^	Japan	Orvil	30	2 (6.7)	1 (3.3)	0 (0)^a^	2011
Marangoni G^87^	United Kingdom	Orvil	13	NA	0 (0)	0 (0)^a^	2012
Jung YJ^48^	South Korea	Orvil	40	NA	2 (5)	1 (2.5)^a^	2013
Liao GQ^88^	China	Orvil	21	NA	1 (4.8)	1 (4.8)^a^	2013
Shim JH^66^	South Korea	Orvil	12	NA	2 (17)	4 (33)^a^	2013
Xie JW^89^	China	Orvil	28	NA	0 (0)	0 (0)^a^	2013
Zuiki T^90^	Japan	Orvil	52	NA	1 (1.9)	11 (21)^a^	2013
Lafemina J^91^	United States	Orvil	17	NA	1 (5.9)	1 (5.9)^a^	2013
Hiyoshi Y^92^	Japan	Orvil	21	NA	2 (9.5)	0 (0)^a^	2014
Ito H^93^	Japan	Orvil	117	NA	2 (1.7)	2 (1.7)^a^	2014
Kwon Y^94^	South Korea	Orvil	36	20 (55.6)	1 (2.8)	2 (5.7)^a^	2014
Wang H^81^	China	Orvil	42	NA	0 (0)	2 (4.8)^a^	2015
Ichikawa D^63^	Japan	Orvil	28	5 (17.9)	0 (0)	1 (3.6)^a^	2015
Lu X^95^	China	Orvil	25	7 (28.0)	0 (0)	0 (0)^a^	2016
Brenkman HJ^96^	Nertherlands	Orvil	47	24 (51.1)	6 (12.8)	11 (23)^a^	2016
Shida A^97^	Japan	Orvil	100	11 (11.0)	4 (4.0)	4 (4)^a^	2016
Yasukawa D^55^	Japan	Orvil	51	5 (9.8)	2 (3.9)	0 (0)^a^	2017
Kawamura H^53^	Japan	Orvil	49	4 (8.2)	2 (4.1)	2 (4.1)^a^	2017
Li X^83^	China	Orvil	19	1 (5.3)	0 (0)	1 (5.3)^a^	2017
Yoshikawa K^56^	Japan	Orvil	36	5 (13.9)	0 (0)	3 (8.3)^a^	2018
Total			840		27 (3.2)	49 (5.8)^a^	

DST, double?stapling technique; HDST, hemi?double stapling technique; NA, not available; PSI, purse?string suture instrument; SST, single?stapling technique.

Orvil, Medtronic plc, Dublin, Ireland.

a *P* < 0.05 (Chi‐square test)

No significant differences were found in these three categories in the frequency of anastomotic leakage. However, the rate of anastomotic stricture was the lowest in SST and the highest in DST/HDST with a transabdominally inserted anvil.

Comparing this result with the review by Inokuchi et al,^50^ our results showed that the occurrence of anastomotic stricture was equivalent in cases of SST reconstruction (2.2% and 2.1%, respectively); however, the incidence rate was lower than that of results in OrVil reconstruction (5.8% and 8.8%, respectively). Standardization of the procedures and recognition of necessary skills for reconstruction using OrVil, including minimization of a small incision through which the tube is pulled out and tension‐free anastomosis, may have contributed to the recent reduction of anastomotic stricture.

In principle, the success of reconstruction mainly depends on sufficient blood supply and tension‐free anastomosis. For sufficient blood supply, the length of detachment from the esophageal stump should be the minimum required for EJS anastomosis. For anastomotic tension, Okata reported that anastomosis under tension significantly increases anastomotic complications.^98^ Some efforts to decrease the tension, such as dissecting a marginal artery of the jejunal artery, sacrificing the jejunum,^99^ and elevating the jejunum through the retrocolic route, should be considered during the operation in the case of EJS under tension.

**Figure 1:**
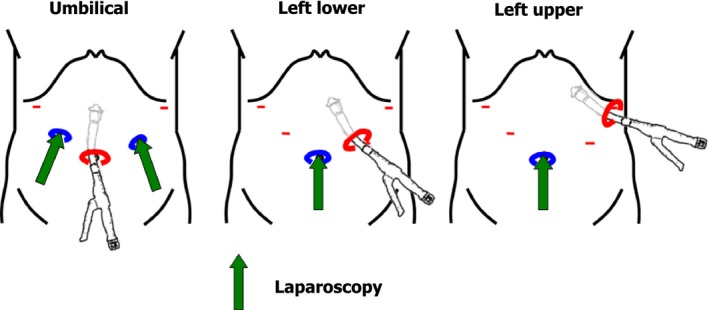
Different approaches of a circular stapler by insertion site. The visual field of the anastomotic plane differs depending on the approach

Next, all of the studies retrieved above were summarized according to the insertion site of the suture instrument, such as left upper, left lower, and umbilical (Table 3).^33,46,48,55,63–65,67–71,73–86,88–90,92,94–97^ As shown in Figure 1, the visual field differed depending on the approach of the anastomotic device, and the left upper abdomen provided the widest visual field of the anastomotic plane.

**Table 3:** Summary reported of esophagojejnostomy insertion site of circular staler n (%)

Authors	Nation	Method	No. of patients	Leakage	Stricture	Year
Left upper						
Omori T^77^	Japan	DST	10	0 (0)	0 (0)	2009
Sakuramoto S^85^	Japan	Orvil	24	0 (0)	0 (0)	2010
Jung YJ^48^	Korea	Orvil	40	2 (5)	1 (2.5)	2013
Du J^69^	China	SST	52	0 (0)	0 (0)	2014
Muguruma K^78^	Japan	DST	32	0 (0)	0 (0)	2014
Hiyoshi Y^92^	Japan	Orvil	21	2 (9.5)	0 (0)	2014
Kwon Y^94^	Korea	Orvil	36	1 (2.8)	2 (5.6)	2014
Ichikawa D^63^	Japan	DST & Orvil	86	0 (0)	3 (3.5)	2015
Yamada T^73^	Japan	SST	10	0 (0)	0 (0)	2015
Lu X^95^	China	Orvil	25	0 (0)	0 (0)	2016
Liu W^76^	china	SST	41	0 (0)	0 (0)	2017
Total			379	5 (1.3)*	6 (1.6)*	
Left lower						
Kachikwu EL^86^	United States	Orvil	16	0 (0)*	3 (18.8)*	2011
Lee JH^33^	South Korea	SST	79	2 (2.5)*	0 (0)*	2012
Kim HI^67^	South Korea	SST	36	0 (0)*	0 (0)*	2013
Kim JH^80^	Korea	DST	58	0 (0)*	1 (1.7)*	2015
Wang H^81^	China	DST & Orvil	84	1 (1.2)*	4 (4.8)*	2015
Amisaki M^74^	Japan	SST	10	0 (0)*	0 (0)*	2016
Ali B^82^	Korea	DST	58	3 (5.2)*	5 (8.6)*	2017
Okuno K^75^	Japan	SST	94	0 (0)*	2 (2.1)*	2017
Total			435	6 (1.4)*	15 (3.4)*	
Umbilical						
Usui S^64^	Japan	SST	15	0 (0)*	0 (0)*	2008
Jeong O^84^	South Korea	Orvil	16	0 (0)*	0 (0)*	2009
Kinoshita T^65^	Japan	SST	10	0 (0)*	0 (0)*	2010
Kunisaki C^46^	Japan	Orvil	30	1 (3.3)*	0 (0)*	2011
Yoshikawa T^68^	Japan	SST	20	0 (0)*	0 (0)*	2013
Liao GQ^88^	China	Orvil	21	1 (4.8)*	1 (4.8)*	2013
Xie JW^89^	China	Orvil	28	0 (0)*	0 (0)*	2013
Zuiki T^90^	Japan	Orvil	52	1 (1.9)*	11 (21)*	2013
Zhao YL^79^	China	DST	26	0 (0)*	0 (0)*	2014
Matsuda T^70^	Japan	SST	21	1 (4.8)*	1 (4.8)*	2015
Kosuga T^71^	Japan	SST & DST	136	9 (6.6)*	17 (12.5)*	2015
Brenkman HJ^96^	Nertherlands	Orvil	47	6 (12.8)*	11 (23.4)*	2016
Shida A^97^	Japan	Orvil	100	4 (4.0)*	4 (4.0)*	2016
Li X^83^	China	DST & Orvil	43	1 (2.3)*	1 (2.3)*	2017
Yasukawa D^55^	Japan	Orvil	51	2 (3.9)*	0 (0)*	2017
Total			616	26 (4.2)*	46 (7.5)*	

DST, double stapling technique; SST, single?stapling technique.

Orvil, Medtronic plc, Dublin, Ireland.

*P* < 0.05 (Chi‐square test).

Results showed that the occurrence rates of anastomotic leakage and stricture were the lowest in the upper left abdomen approach, and anastomotic complications were significantly higher in a midline umbilical approach. These results suggested that a good visual field may reduce anastomotic complications, and surgeons should be particularly attentive to maintaining a good visual field for the anastomotic plane, even in the umbilical approach, and avoiding unnecessary tension during anastomosis.

The flexible laparoscope should be useful in obtaining a good view where a straightforward view is difficult. However, only a few reports were confirmed to use the flexible laparoscope in each approach; therefore, it is not yet clear whether these complications can be reduced by the use of a flexible scope.

## 6 CONCLUSION

We reviewed several recent reports on lymphadenectomy and reconstruction in LTG. As cancers located at the upper third of the stomach and at the esophagogastric junction have increased in recent years,^100^ in the future, safe and secure LTG is important. According to the Japanese gastric cancer treatment guidelines, LTG for clinical stage I gastric cancer may be carried out; however, it is recommended that the procedure be conducted under the guidance of experienced surgeons. In contrast, LTG has been rated by the guidelines of the Japan Society for Endoscopic Surgery (2014) as recommendation C1 (may be considered for a patient in need of total gastrectomy, but no scientific evidence in support of the procedure is currently available). Those who consider challenging the procedure should plan to do so with sufficient caution as postoperative complications were reported to occur significantly more often in the first year of its introduction.^24^

Concerning advanced cancer, a Korean group has launched a large multi‐institutional clinical study for prognostic evaluation of lymph node dissection in LTG for advanced gastric cancer (KLASS‐06). LTG for advanced gastric cancer should be carried out on a trial basis until the definitive results are available, and surgeons should be particularly attentive to nodes No. 10 and 11d in a lymphadenectomy without lessening the quality of lymph node dissection compared with OTG.

Then again, inappropriate reconstruction sometimes results in postoperative complications, some of which have recently been reported to correlate with poor long‐term oncological outcome.^101^, ^102^ In that sense, surgeons must give scrupulous attention to leakage and stricture after EJS while understanding the advantages and disadvantages of each anastomotic device and approach.

## DISCLOSURE

Conflict Of Interest: Authors declare no conflict of interests for this article.
